# Clinical value of bundled care protocols in enhancing postoperative recovery among lung cancer patients

**DOI:** 10.12669/pjms.41.11.12304

**Published:** 2025-11

**Authors:** Cuifang Liu, Pei Liu, Hongyu Liang, Kun Wang, Xiaoxue Huang, Yaqian Li

**Affiliations:** 1 Cuifang Liu, Department of Thoracic Surgery, Affiliated Hospital of Hebei University, Baoding 071000, Hebei, China; 2 Pei Liu, Department of Thoracic Surgery, Affiliated Hospital of Hebei University, Baoding 071000, Hebei, China; 3 Hongyu Liang, Department of Thoracic Surgery, Affiliated Hospital of Hebei University, Baoding 071000, Hebei, China; 4 Kun Wang, Department of Thoracic Surgery, Affiliated Hospital of Hebei University, Baoding 071000, Hebei, China; 5 Xiaoxue Huang, Department of Thoracic Surgery, Affiliated Hospital of Hebei University, Baoding 071000, Hebei, China; 6 Yaqian Li, Department of Thoracic Surgery, Affiliated Hospital of Hebei University, Baoding 071000, Hebei, China

**Keywords:** Bundled care, Lung cancer, Postoperative recovery, Clinical value

## Abstract

**Objective:**

To evaluate the clinical value of bundled care protocols in enhancing postoperative recovery among lung cancer patients.

**Methodology:**

This was a retrospective study. A total of 120 patients undergoing radical lung cancer surgery in the thoracic surgery department of Affiliated Hospital of Hebei University from November 2022 to November 2024 were enrolled randomly assigned to the study group and the control group using a random number table (n=60 each group). The control group received routine nursing care, while the study group was managed using a bundled care protocol. Indicators including pulmonary function parameters, immune status, postoperative complication rates, quality-of-life scores and satisfaction levels were compared between the two groups before and after the intervention.

**Results:**

Post-intervention, the study group demonstrated significant improvements in FEV1, FVC, FEV1/FVC and PEF values compared to the control group(P=0.00). Immune indicators, including CD3^+^, CD4^+^ and CD4^+^/CD8^+^, in the study group were also markedly higher than those in the control group(P=0.00). The incidence of postoperative complications was 8% in the study group, markedly lower than the 23% observed in the control group (P=0.02). Post-intervention, KPS scores and SF-36 quality-of-life scores were significantly higher in the study group compared to the control group(P=0.00). Patient satisfaction in the study group reached 100%, compared to 90% in the control group, with a statistically significant difference(P=0.01).

**Conclusion:**

Bundled care protocols demonstrate significant clinical benefits for lung cancer patients undergoing surgical treatment, including enhanced pulmonary function recovery, improved immune reconstitution, reduced postoperative complication rates and elevated quality of life and care satisfaction.

## INTRODUCTION

Lung cancer remains a prevalent malignancy with high incidence and mortality rates, accounting for 11.4% of all cancers. According to recent statistics, approximately 2.2 million new cases of lung cancer were diagnosed globally in 2020, with an estimated 1.796 million deaths, representing 18% of all cancer-related deaths.^1^ Research^2^ indicate that lung cancer is the leading cause of cancer-related deaths and ranks as the second most common malignancy in Western countries. Lung cancer has thus emerged as a critical public health concern that poses substantial threats to global health and well-being, with its prevention and therapeutic management becoming a primary focus of contemporary research efforts.^3^ Radical lung cancer surgery remains the primary treatment modality for lung cancer.^4^

Surgery, being inherently invasive, predisposes patients to a spectrum of postoperative complications including respiratory dysfunction, immunosuppression and other surgery-related effects. These complications not only extend the duration of hospitalization but also exacerbate the financial burden on patients and significantly impair their quality of life. Enhancing the quality of postoperative care for patients undergoing radical lung cancer surgery has always been a critical focus in clinical practice. Bundled care, initially conceptualized by the Institute for Healthcare Improvement (IHI) in the United States, is designed to assist healthcare professionals in delivering optimal medical and nursing interventions. It is not simply a combination of measures, but rather an evidence-based methodology comprising a set of clinically validated interventions that have been demonstrated to improve patient outcomes.^5^

Typically comprising three to five elements, bundled care is characterized by its holistic nature, synergistic implementation of multiple interventions, enhanced effectiveness, greater care reliability and ability to achieve homogeneity in nursing quality.^6^ Bundled care was initially introduced into the field of nursing to prevent ventilator-associated pneumonia. However, with continued research domestically and internationally, its application has expanded to various clinical domains in recent years, including the management of critically ill patients, patients with malignancy, those with central venous catheter placements or drainage tubes, pressure ulcer patients and individuals recovering from traumatic brain injuries. Studies indicate that implementing bundled care significantly improves clinical outcomes and enhances patients’ quality of life.^7^ This study aimed to explore the effectiveness of bundled care protocols in enhancing postoperative recovery among lung cancer patients and evaluate the intervention’s outcomes.

## METHODOLOGY

This was a retrospective study. A total of 120 patients undergoing radical lung cancer surgery in the thoracic surgery department of Affiliated Hospital of Hebei University from November 2022 to November 2024 were enrolled and randomly assigned to either the study group or the control group using a random number table, with 60 cases in each group. According to the data of each indicator in the pre-survey, the sample size is estimated by 95% confidence interval, and the largest one is the sample size of the study. The sample size required for each group was ≥60 cases on the basis of Fisher exact probability. The control group received routine nursing care in thoracic surgery, while the study group was managed using a bundled care protocol based on standard care, with a tailored nursing intervention plan for postoperative recovery. In the study group, there were 33 males and 27 females, aged 42-67 years, with an average age of 53.62 ± 4.70 years. In the control group, there were 35 males and 25 females, aged 43-66 years, with an average age of 54.28 ± 4.42 years. The general data of both groups showed no significant differences, indicating comparability between the groups (Table-I).

**Table-I:** Comparative analysis of general data between the study group and the control group (*χ̅*±*S*) n=60.

Indicators	Study Group	Control Group	t/χ^2^	P
Age (years)	53.62±4.70	54.28±4.42	0.79	0.43
Gender (male)	33 (55%)	35 (58%)	0.14	0.71
Pathological types				
Adenocarcinoma (cases, %)	41 (68%)	40 (67%)	0.04	0.85
Squamous carcinoma (cases, %)	12 (20%)	14 (23%)	0.20	0.66
Others (cases, %)	7 (12%)	6 (10%)	0.09	0.77
Surgical Methods			0.17	0.68
Unilateral pneumonectomy (cases, %)	17 (28%)	15 (25%)		
Lobectomy (cases, %)	43 (72%)	45 (75%)		
Marital status			0.77	0.38
Married (cases, %)	52 (87%)	55 (92%)		
Unmarried (cases, %)	8 (13%)	5 (8%)		
Education level				
Below Middle School	10 (17%)	15 (25%)	1.26	0.26
Middle School	34 (57%)	30 (50%)	0.54	0.46
College and Above	16 (26%)	15 (25%)	0.04	0.84

P>0.05.

### Ethical Approval:

The study was approved by the Institutional Ethics Committee of Affiliated Hospital of Hebei University (No.: HDFYLL-IIT-2023-036; Date: March 31, 2024) and written informed consent was obtained from all participants.

### Inclusion criteria:

- Patients with a confirmed clinical diagnosis of lung cancer who underwent radical surgery.^8^
- Those aged 40~70 years.
- Those with complete clinical records available.
- Those with clear consciousness, absence of psychiatric disorders and the ability to actively participate in treatment and nursing interventions.
- Those who provided written informed consent.
- Obtained the informed consent of participants’ families.
- Those without other malignancies or significant neuropsychiatric symptoms.

### Exclusion criteria:

- Patients with poor general condition accompanied by severe liver and kidney dysfunction or other organic diseases.
- Those diagnosed with other malignancies or immune-related disorders.
- Those with poor compliance, inability to complete the planned protocol, non-compliance with follow-up, or the presence of mental illnesses or cognitive impairments.
- Those with metastatic cancer and an anticipated survival period of less than one year.

### Methods:

The control group received routine nursing care, including health education, an introduction to the hospital environment, disease-related knowledge, precautions for surgical treatment and guidance on healthy behavior. Patients were educated on postoperative training and encouraged to follow a structured rehabilitation plan. Following discharge, a six-month follow-up was conducted through a combination of telephone consultations and outpatient visits to assess the patients’ adherence to rehabilitation exercises.

In addition to the routine care provided to the control group, the study group received a bundled care protocol. The protocol included health education, chest drainage tube care, respiratory function exercises and physical exercises.

#### Health Education:

Patients were informed about preoperative preparations, the purpose of medications, surgical procedures and fasting times. The bundled care team emphasized the importance and objectives of postoperative functional exercises, providing detailed instructions on rehabilitation knowledge and skills to enhance patient motivation, initiative and compliance.

#### Respiratory function exercises:

Patients were instructed to perform calm breathing, forward-leaning exhalation and upright inhalation for 5-10 minutes per session.

#### Chest drainage tube care:

This prevention involved observing the color, nature and volume of the drainage fluid and squeezing the drainage tube hourly to maintain patency.

Postoperative physical activity guidance was tailored to the patient’s condition. Patients were advised to avoid breath-holding, straining during bowel movements, excessive coughing and intense physical activity on the day the chest drainage tube was removed. On postoperative day one, patients engaged in bedside activities for 3-5 minutes from days two to three, they walked 5-10 minutes three times daily. From day four until discharge, they walked 10-20 minutes once daily and climbed 10 flights of stairs twice daily, with minimal rest periods. Post-discharge, patients were advised to exercise for 10-20 minutes twice daily for the first two weeks. From two weeks to one-month post-discharge, the exercise duration was increased to 20-30 minutes twice daily, with options including walking, stair climbing, or hiking. To ensure safe and effective exercise intensity, patients were trained to monitor their pulse during physical activity. Regular follow-up was conducted to encourage adherence to undergo exercise training. All operations were performed by the same group of doctors.

### Observation Indicators:

- Comparison of pulmonary function indicators before and after intervention in both groups: forced expiratory volume in the first second (FEV1), forced vital capacity (FVC), the ratio of FEV1 to FVC (FEV1/FVC) and peak expiratory flow rate (PEF) were measured using a Belgian Medisoft pulmonary function analyzer.
- Comparative analysis of immune function status: Peripheral blood samples (3 mL) were collected from both groups of patients on an empty stomach before and after the intervention. After centrifugation at 4000 rpm (radius: 5 cm) for 10 min, the plasma on top was harvested, venous blood was collected before and after the intervention to measure T lymphocyte subsets, including CD3^+^, CD4^+^, CD8^+^ and CD4^+^/CD8^+^, with comparative analysis of the changes in these indicators.
- Comparison of the incidence of surgical complications between the two groups: postoperative complications assessed included atelectasis, pulmonary infection, lower-extremity venous thrombosis and arrhythmia.
- Quality of life scores: changes in quality of life were evaluated before and after intervention using the Karnofsky Performance Status (KPS) scale and the SF-36 Quality of Life scoring system.^9^,^10^
- Patient satisfaction was assessed using the Patient Satisfaction Questionnaire Short Form (PSQ-18),^11^ with satisfaction rates categorized as very satisfied, fairly satisfied, satisfied, uncertain, or dissatisfied. Total satisfaction rate = (very satisfied + fairly satisfied + satisfied)/total cases × 100%.

### Statistical Analysis:

All data were statistically analyzed using the SPSS 20.0 software (SPSS Inc., Chicago, IL, USA). Measurement data were presented as mean ± standard deviation (*χ̅*±*S*). Between-group comparisons were conducted with t-tests and rate comparisons were performed using chi-square (χ²) tests. A *P*-value <0.05 was considered a statistically significant difference.

## RESULTS

Before intervention, there were no statistically significant differences in FEV1, FVC, FEV1/FVC, or PEF values between the two groups(P>0.05). After intervention, all these indicators improved in both groups, with significantly greater improvements observed in the study group than in the control group (P=0.00) (Table-II).

**Table-II:** Comparison of Pulmonary Function Indicators Before and After Intervention Between the Two Groups (*χ̅*±*S*), n=60

Indicator		Study Group	Control Group	t	p
FEV_1_ (L)	Before Intervention	1.53±0.14	1.54±0.20	0.32	0.75
	After Intervention*	2.57±0.16	2.38±0.18	6.11	0.00
FVC (L)	Before Intervention	1.93±0.45	1.92±0.31	0.14	0.89
	After Intervention*	2.60±0.31	2.07±0.15	9.36	0.00
FEV_1_/FVC (%)	Before Intervention	43.56±5.27	43.31±5.14	0.26	0.79
	After Intervention*	58.42±4.76	52.67±4.23	7.25	0.00
PEF (L/min^-1^)	Before Intervention	185.50±30.17	183.83±28.97	0.38	0.71
	After Intervention*	213.46±31.42	197.33±30.85	2.87	0.00

* P<0.05.

Before intervention, there were no significant differences between the two groups in levels of CD3^+^, CD4^+^, CD8^+^, or CD4+/CD8^+^ (P>0.05). After intervention, improvements were observed in CD3^+^, CD4^+^, CD8^+^ and CD4+/CD8^+^ in both groups, with significantly greater improvements in the study group (P=0.00). CD8^+^ levels showed no significant changes before and after treatment in either group (P>0.05) (Table-III).

**Table-III:** Comparison of T Lymphocyte Subsets Before and After Intervention Between the Two Groups (*χ̅*±*S*), n=60.

Indicator		Study Group	Control Group	t	P
CD3^+^ (%)	Before Intervention	42.18±5.62	42.31±5.43	0.13	0.90
	After Intervention*	49.21±7.35	45.20±7.16	3.03	0.00
CD4^+^ (%)	Before Intervention	23.29±6.44	23.17±6.42	0.10	0.92
	After Intervention*	35.16±7.31	28.53±6.98	5.08	0.00
CD8^+^ (%)	Before Intervention	23.15±5.26	23.30±5.11	0.16	0.87
	After Intervention	24.61±6.27	24.57±6.09	0.04	0.97
CD4^+^/CD8^+^	Before Intervention	1.22±0.20	1.21±0.18	0.29	0.77
	After Intervention*	1.84±0.21	1.63±0.13	6.58	0.00

* P<0.05.

The incidence of surgical complications was significantly lower in the study group (8%) compared to the control group (23%), with a statistically significant difference (P=0.02) (Table-IV). Before intervention, there were no significant differences between the two groups in the KPS scores and SF-36 quality of life scores (P>0.05). After intervention, the above indicators increased for both groups, with the study group showing a more significant increase compared to the control group and the difference was statistically significant (P=0.00) (Table-V). The patient satisfaction rate was 100% in the study group and 90% in the control group, with the study group significantly higher than the control group and the difference was statistically significant (P=0.01) (Table-VI).

**Table-IV:** Comparative Analysis of Incidence Rates of Surgical Complications Between the Two Groups (*χ̅*±*S*), n=60.

Group	Atelectasis	Pulmonary Infection	Lower Limb Venous Thrombosis	Arrhythmia	Incidence Rate
Study Group	1	2	0	2	5 (8%)
Control Group	4	3	3	3	14 (23%)
χ^2^					5.06
P					0.02

P<0.05.

**Table-V:** Comparative Analysis of Quality-of-Life Scores Before and After Treatment Between the Two Groups (*χ̅*±*S*), n=60.

Indicator		Study Group	Control Group	t	p
KPS Score	Before Intervention	63.80±7.44	64.13±7.18	0.25	0.81
	After Intervention*	72.75±8.36	68.35±8.14	2.92	0.00
SF-36 Score	Before Intervention	46.57±7.63	46.82±7.55	0.18	0.86
	After Intervention*	64.31±8.84	60.21±7.66	2.71	0.00

* P<0.05.

## DISCUSSION

Our findings demonstrated that the overall incidence of postoperative complications was significantly lower in the study group following bundled care compared to the control group (P=0.02), consistent with similar findings by Dean HF et al.^12^ In contrast to conventional nursing measures, the bundled nursing care strategy proved completer and more targeted, effectively reducing the incidence of postoperative complications and improving clinical outcomes. Additionally, our study revealed a more pronounced improvement in KPS scores and SF-36 quality of life scores in the study group post-intervention compared to the control group (P=0.00). Post-intervention immunological parameters, including CD3^+^, CD4^+^ and CD4^+^/CD8^+^, improved in both groups, with significantly greater enhancements observed in the study group, achieving statistical significance (P=0.00).

Moreover, patient satisfaction in the study group reached 100%, notably higher than the 90% satisfaction rate in the control group (P=0.01). The bundled nursing care strategy, characterized by its efficiency and quality, has demonstrated substantial efficacy in postoperative care across various diseases. Reports^13^ indicate that the introduction of bundled care principles in the postoperative care of patients with gastric cancer, ovarian cancer and thyroid cancer has offered new clinical insights, effectively promoting recovery and enhancing quality of life. Furthermore, it has been shown to improve psychological resilience, self-efficacy, immune function and overall satisfaction with nursing care.

**Table-VI:** Comparative Analysis of Patient Satisfaction Between the Two Groups (*χ̅*±*S*), n=60.

Group	Highly Satisfied	Quite Satisfied	Satisfied	Uncertain	Not Satisfied	Overall Satisfaction *
Study Group	50	6	4	0	0	60 (100%)
Control Group	30	20	4	4	2	54 (90%)
χ^2^						5.22
P						0.01

* P<0.05.

Our results also showed that the study group exhibited significant improvements in FEV1, FVC, FEV1/FVC and PEF values post-intervention compared to the control group, with differences reaching statistical significance (P=0.00). These outcomes can be attributed to essential components of the bundled nursing care strategy, such as encouraging and guiding patients to expectorate appropriately, promptly clearing respiratory secretions and improving pulmonary compliance through increased expiratory flow rates. Nutritional support improved patients’ nutritional status, while enteral nutrition strategies helped prevent gastroesophageal reflux-induced pulmonary infection. Respiratory training management, combined abdominal-thoracic exercises and diaphragmatic pressure generation replaced respiratory muscle function, enhancing pulmonary ventilation and facilitating secretion clearance.^14^ This approach promoted vital capacity training, preventing pulmonary complications. Passive and active limb exercises contributed to the recovery of various body functions, improved physical activity and resilience and further enhanced autonomous sputum expectoration capabilities.^15^ Altogether, these measures significantly improved overall therapeutic outcomes.

Lung cancer is one of the most commonly diagnosed malignancies in clinical practice, characterized by persistently high incidence and mortality rates that show a trend of gradual increase over time.^16^ It predominantly affects middle-aged and elderly populations, with a higher incidence in men compared to women. Common symptoms include low-grade fever, chest pain, cough, sputum production and dyspnea,^17^ severely compromising patients’ physical and mental health. At present, surgical intervention remains the first-line treatment for lung cancer and is the only curative option available.^18^ However, in clinical practice, most patients experience significant physiological and psychological stress responses to surgery, resulting in anxiety and fear.

Postoperative complications such as pulmonary dysfunction, prolonged bed rest, increased respiratory secretion due to surgical trauma and associated risks like atelectasis, pulmonary infections and deep vein thrombosis are also common, significantly impairing patients’ prognosis and quality of life.^19^ Clinical studies have demonstrated that negative emotional states can induce neuroendocrine alterations, diminishing immune function and reducing surgical tolerance, thereby adversely impacting the safety of surgery and postoperative recovery. These negative effects are particularly pronounced in elderly patients.^20^ Accordingly, providing appropriate nursing interventions to surgical patients to alleviate stress responses and enhance their acceptance and cooperation with surgical treatment is essential for improving prognostic outcomes and holds significant clinical value.

In conventional nursing care for lung cancer surgical patients, standard interventions have traditionally been employed. While these interventions encompass fundamental aspects such as admission assessment, preoperative care, vital sign monitoring and routine measures essential for ensuring surgical success, they often fall short in addressing patients’ comprehensive needs. Conventional care frequently neglects psychological support, functional recovery and rehabilitation, limiting its effectiveness in mitigating postoperative complications and prognostic risks. Clustered nursing, guided by evidence-based medicine, provides systematic and targeted nursing support for specific diseases based on verified evidence, thereby delivering high-quality care.

This approach has been widely applied across various clinical disciplines and diseases, yielding favorable outcomes.^21^ Introducing bundled nursing interventions in the postoperative care of lung cancer patients enables the delivery of superior, more professional and comprehensive care tailored to patients’ specific conditions and recovery requirements. It facilitates patient and family health education to enhance understanding of the disease and the nursing plan, alleviates negative emotions such as anxiety and fear and improves clinical cooperation. Furthermore, establishing nursing teams to create individualized nursing profiles and evaluate postoperative risks ensures meticulous care.

### Limitations:

The limitations of our study include a small sample size and a short follow-up duration. Future clinical work will focus on expanding the sample size and extending follow-up periods to more objectively evaluate the advantages and disadvantages of this intervention protocol, benefiting a larger cohort of patients.

## CONCLUSIONS

Implementing a bundled nursing care strategy for lung cancer surgical patients promotes postoperative pulmonary function recovery, reduces the incidence of surgical complications, enhances quality of life and improves immune status and nursing satisfaction. These advantages significantly accelerate recovery and underscore its important clinical value, warranting broader application.

### Authors’ Contributions:

**CL** and **PL:** Study design, literature search and manuscript writing.

**HL** and **KW:** Data collection, data analysis, interpretation and critical review.

**XH** and **YL:** Manuscript revision and validation, critical analysis.

All authors have read, approved the final manuscript and are responsible for the integrity of the study.
